# Fixation strength in arthroscopic labral repair of the hip: A head-to-head comparison of the biomechanical performance of a biocompatible vs. all-suture anchor in the setting of acetabuloplasty

**DOI:** 10.1371/journal.pone.0293738

**Published:** 2023-11-02

**Authors:** Benton A. Emblom, Brian L. Walters, Logan E. Mast, David P. Beason, John A. Ruder, Michael K. Ryan, Stephen A. Gould, Martin L. Schwartz

**Affiliations:** 1 American Sports Medicine Institute, Birmingham, Alabama, United States of America; 2 Andrews Sports Medicine and Orthopaedic Center, Birmingham, Alabama, United States of America; 3 Radiology Associates of Birmingham, Birmingham, Alabama, United States of America; University of Victoria, CANADA

## Abstract

Much is known about the biomechanical performance of various types of suture anchors commonly used for labral fixation in the shoulder; however, similar studies in the hip are less common. We sought to compare all-suture and polyether ether ketone small-diameter anchors in the setting of labral repair during hip arthroscopy, with and without acetabuloplasty. We hypothesized that the biomechanical properties of the all-suture group when compared to polyether ether ketone anchors would be similar amongst native acetabula and significantly less following acetabuloplasty and that pullout forces would be reduced in the anterior and inferior regions of the acetabulum compared to the superior region. Bone density was measured in nine matched pairs of fresh-frozen cadaveric acetabula in the superior, anterosuperior, and anterior regions. Acetabuloplasty was performed in all three regions, while the contralateral acetabulum was left in situ as a control. Suture anchors were placed such that one each of two different types was placed within each region. Specimens were tested in cyclic fatigue and loaded to failure. The all-suture group had significantly higher cyclic displacement compared to the polyether ether ketone, but there was no significant difference in ultimate load, regardless of acetabuloplasty. Amongst all non-resected specimens, the lowest bone density was observed consistently in the inferior region. Our results indicate that, with or without acetabuloplasty, a small-diameter polyether ether ketone anchor appears to be more stable than an all-suture anchor, which needs to be set first.

## Introduction

Femoroacetabular impingement (FAI) is a well-known source of hip pain [1]. The type of lesion dictates the type of damage that results from the FAI [2]. Pincer lesions are the results of bony overgrowth on the acetabulum and result in labral damage [2]. Cam lesions are the result of bony overgrowth on the femoral neck and result in intraarticular pathology [2]. While traditionally, FAI was treated with an open surgical procedure, advancements in arthroscopic techniques have allowed this pathology to be treated arthroscopically with similar results [3].

Treatment options for labral damage include debridement, resection, reconstruction, and repair using suture anchors [4]. Both clinical and basic science studies favor labral repair versus labral debridement [5]. Due to a better understanding of the labrum’s ability to heal, the labrum is now often repaired using suture anchors [6]. While large solid anchors have performed well, they have a large footprint and require significant bone loss at its insertion site, and in certain locations are at risk for joint penetration.

Smaller anchors have been developed in an attempt to reduce that amount of bone removed at the anchor’s insertion site. One such anchor is the all-suture anchor. Compared with their solid-anchor counterparts, these anchors require a smaller pilot hole and have demonstrated good results clinically when used for acetabular labral repair. The all-suture anchors contain one or more ultra-high-molecular-weight-polyethylene (UHMWPE) suture that passes through a sleeve or tape that functions as the anchor. Once deployed, the application of tension to the suture increases the diameter of the anchor, allowing it to securely lock beneath the cortical surface while decreasing the amount of bone lost in the process; however, if the anchor does not deploy correctly, it is prone to not fully expand and slip through the pilot hole before locking under the cortical surface. There is also a concern of clinical failure secondary cyst formation and subsequent tunnel widening [7, 8].

The primary purpose of this study was to compare the cyclic and load to failure properties of an all-suture anchor and a polyether ether ketone (PEEK) anchor in human cadaveric acetabula with and without acetabuloplasty. The authors hypothesized that the biomechanical performance of the all-suture anchor would be inferior following the acetabuloplasty when compared to the solid anchor.

## Materials and methods

A single PEEK anchor and a single all-suture anchor were tested in this study. Eighteen fresh-frozen cadaveric acetabula (9 matched pairs) from donors (6 female, 3 male) under 60 years of age (mean of 51±6 years, range of 44–58 years) were used for this study. Institutional review board approval was not required for our study using tissue from non-identifiable donors; however, taped and/or written informed consent was obtained from the donor (or appropriate authorizing party) by the tissue vendor (MedCure, Portland, OR). Each specimen underwent computed tomography (CT) imaging using a GE LightSpeed VCT 64 slice scanner (GE Medical, Milwaukee, WI, USA) to assess bone mineral density. Quantitative CT-assisted osteodensitometry provided three-dimensional volumetric analysis of acetabular bone density. Relative bone density was measured in Hounsfield units (HU) of each CT slice. Hounsfield units provide a density scale where the X-ray absorption of water is given the value of zero HU and air the value of -1000 HU. This allowed for analysis of relative bone density in the various zones of the acetabulum as described below. Following CT scans, each cadaveric specimen was dissected and stripped free of all soft tissue except for the labrum, which was left intact. Each specimen was then potted in polymethyl methacrylate (PMMA) bone cement with a drywall screw inserted into the acetabular fossa to act as an anchor.

**Fig 1:**
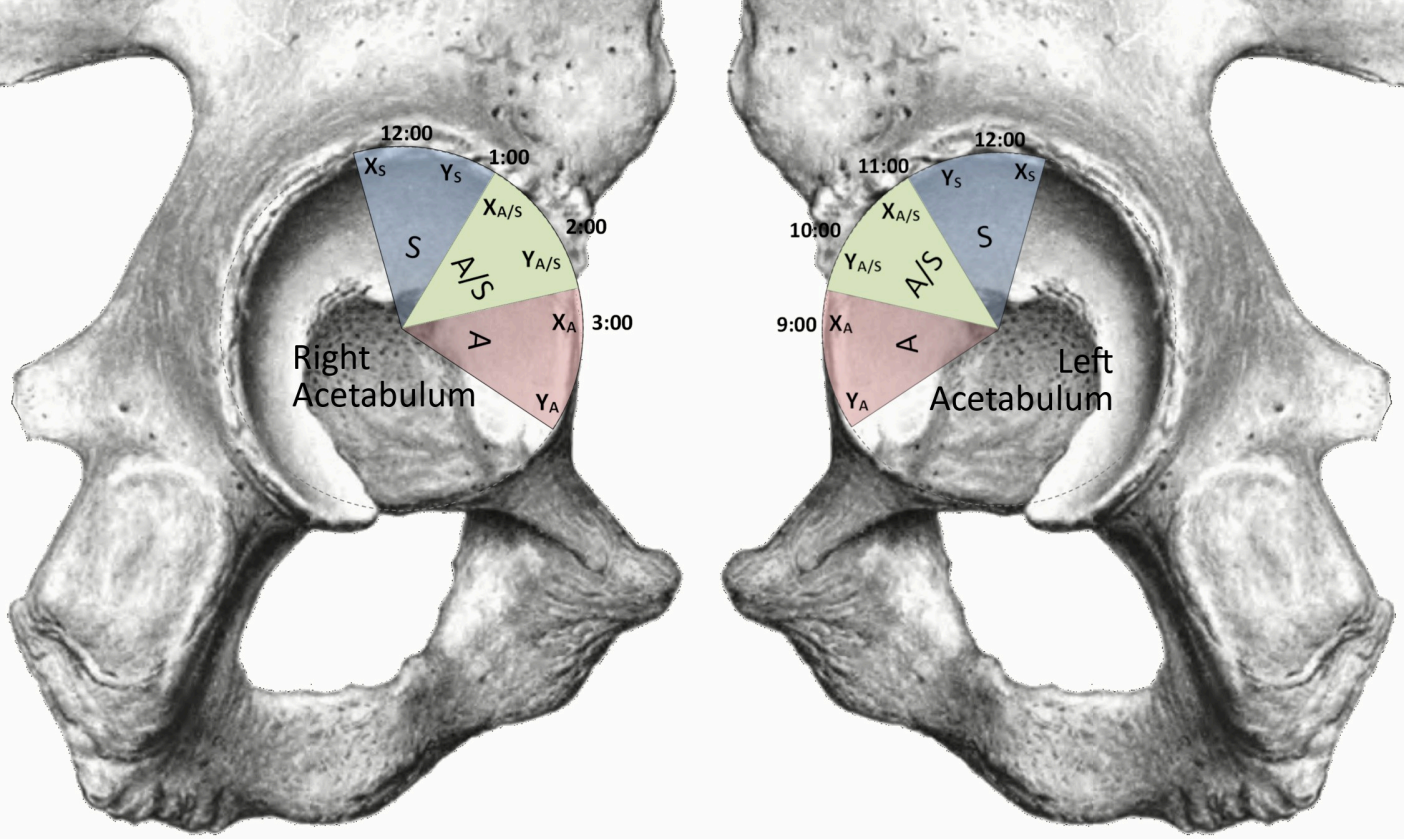
Schematic of acetabular regions. One side received a 3 mm bony decortication; the contralateral side did not. Note that X represents the all-suture anchors; Y represents the PEEK anchors. Regions S, A/S, and A represent the superior (blue), anterosuperior (green), and anterior regions (red), respectively. Regions are shown relative to their position on the clock face.

Each acetabulum was partitioned into the three areas of interest (anterior, anterosuperior and superior). The anchors were rotated between the sites to achieve a consistent comparison. The regions corresponded on the clock face to 11:30–4:00 on a right specimen and 8:00–12:30 on a left specimen (Fig 1). These regions were defined by sections of a clock face, specifically 11:30–1:00 (superior), 1:00–2:30 (anterosuperior), and 2:30–4:00 (anterior) on a right specimen, and 11:00–12:30 (superior), 9:30–11:00 (anterosuperior), and 8:00–9:30 (anterior) on a left specimen. One specimen from each matched pair had an acetabuloplasty (i.e., decorticated group) performed using an Acromionizer 5.5 mm burr (Smith & Nephew, Memphis, TN, USA) to resect approximately 3 mm of the acetabular rim from each of the three regions of interest; the contralateral matched pair served as a non-decorticated control. The determination of which side underwent resection, and which served as control was determined by random number generation.

**Fig 2:**
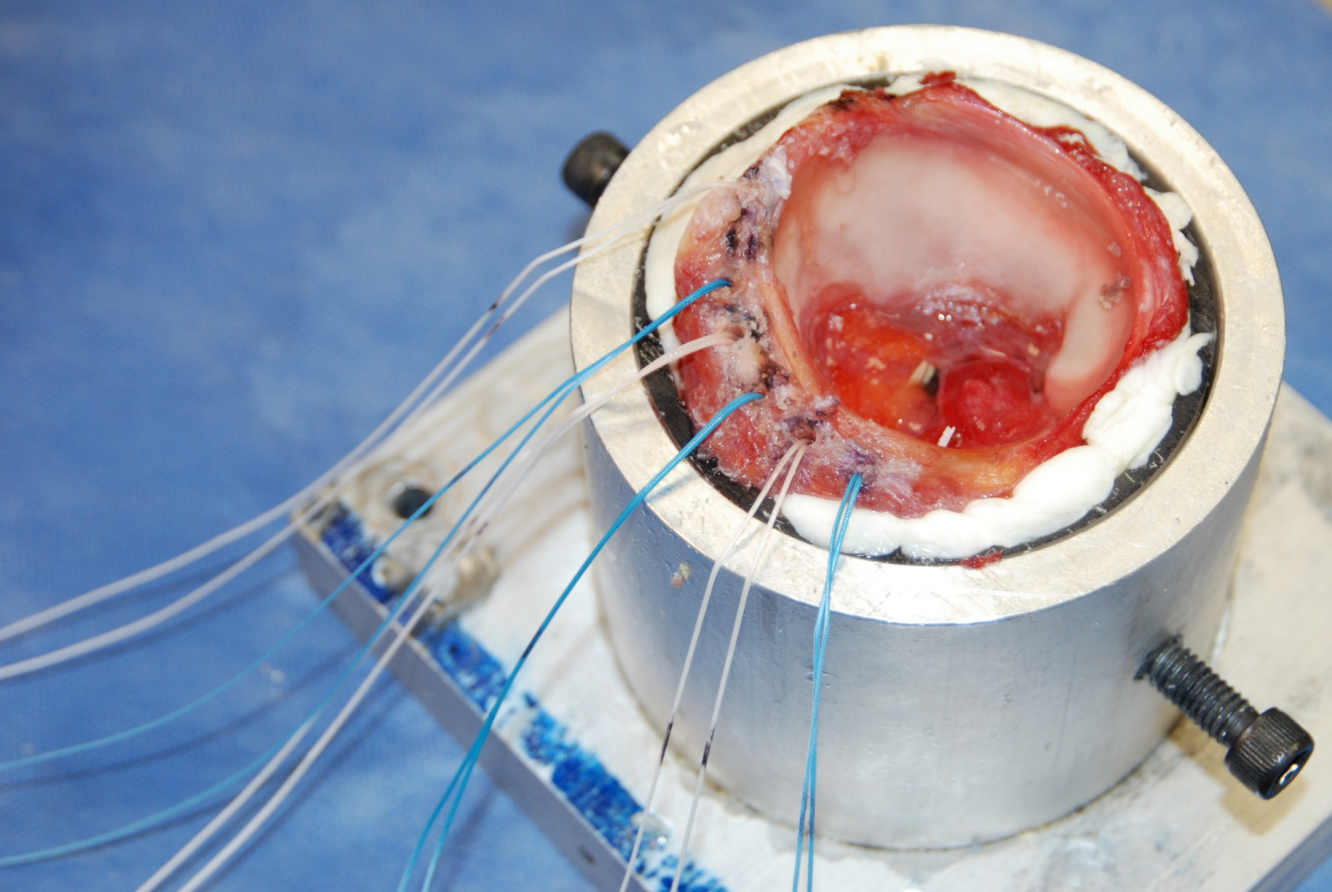
Potted acetabulum in base fixture. The labrum contained three all-suture anchors and three PEEK anchors.

Two anchors intended for acetabular labral repair were used in this study. The BioRaptor 2.3 PEEK anchor loaded with a single Ultrabraid #2 Suture (Smith & Nephew, Memphis, TN, USA) and the SutureFix Ultra 1.7 mm XL All-Suture Anchor loaded with a single Ultrabraid #2 Suture (Smith & Nephew, Memphis, TN, USA). All anchors were inserted by one of two sports medicine fellowship trained orthopaedic surgeons. Anchors were inserted according to the manufacturer’s recommendations. A curved drill guide was used to minimize the risk of subchondral and psoas tunnel perforation [9]. The anchors were spaced 10 mm apart to ensure the bony bridge between anchors was not compromised by crack propagation during the testing [10]. One of each type of anchor was placed within each of the three regions (Fig 2).

**Fig 3:**
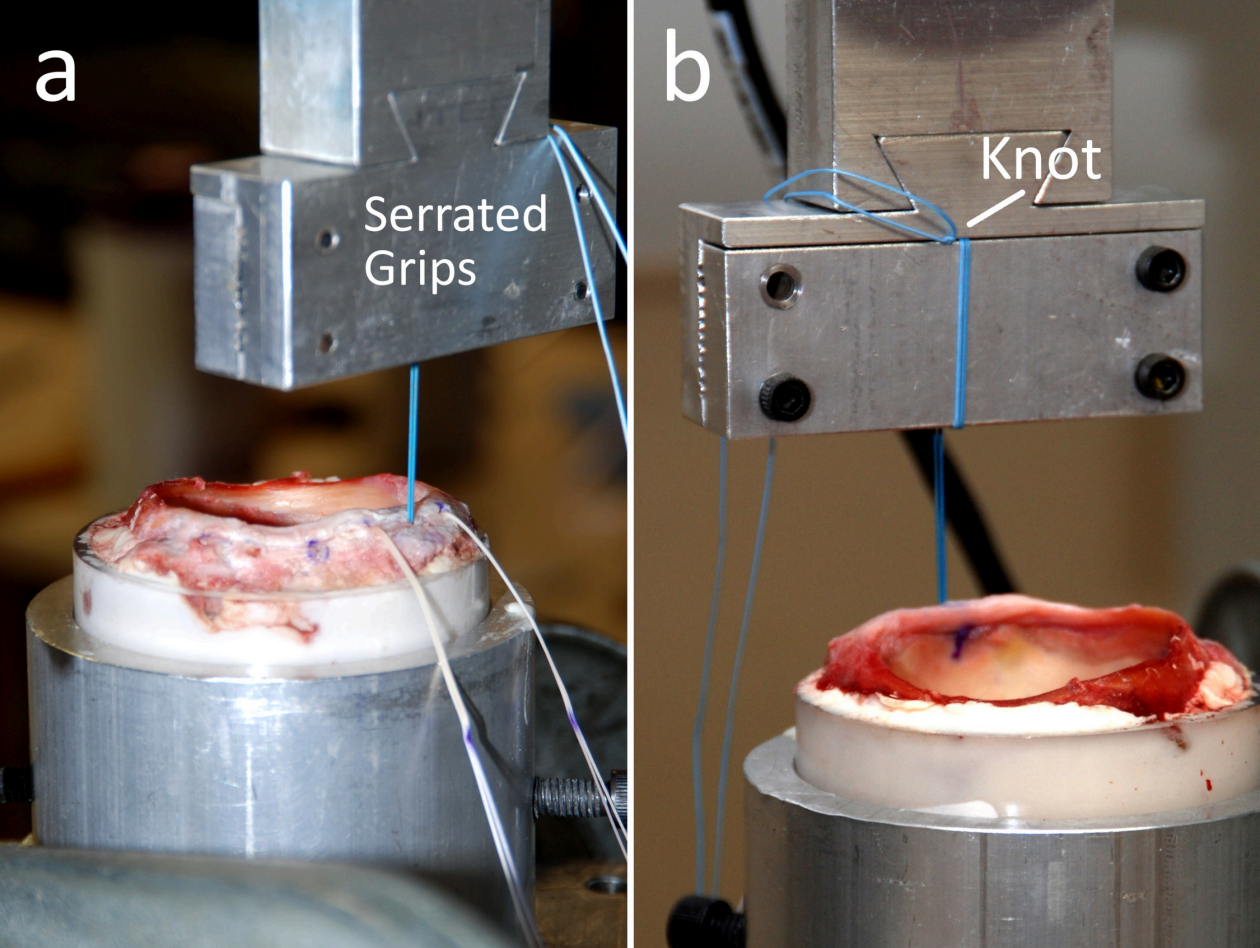
Mechanical testing setup. (a) Rear and (b) front views, showing a suture anchor implanted in the potted acetabulum and loaded in tension within the mechanical test frame.

The potted acetabula were positioned in an MTS 858 MiniBionix mechanical test frame (MTS Systems, Eden Prairie, MN, USA) using custom fixtures. Sutures were gripped at a consistent distance (33-mm gauge length) away from the anchor to eliminate effects of suture elasticity between specimens and tensioned in line with the angle of anchor insertion (Fig 3A). The suture strands were wrapped around the face of the clamp prior to the clamp being tightened, resulting in them being double clamped within the grips. Further, a double knot was tied at the grip opening to create an obstruction, ensuring that there was no slippage within the grips during the test (Fig 3B). The constructs were preloaded to 10 N at 1 N/s, and this preload was held for 60 seconds. After preloading, the constructs were cycled from 10 to 60 N at 1 Hz for 500 cycles [10]. Anchors that remained intact following cycling were then subjected to a single load to failure conducted at 3 mm/min. Data were collected using MTS TestStar IIs software. Displacement after 20, 200, and 500 cycles, along with ultimate load to failure were recorded. Clinical failure was defined as displacement greater than 5 mm during cyclic loading, this has been thought to be clinically relevant during biomechanical testing [11]. Catastrophic failure was defined as anchor pullout during cyclic loading.

For analysis of continuous variables, a two-way repeated measures analysis of variance (ANOVA) test was used to compare means. For each analysis, significance was set at p < 0.05. A post-hoc Tukey’s HSD (honest significant difference) was performed to allow pairwise comparison. All statistical analyses were performed using JMP 10.0.0 (JMP, Cary, NC).

## Results

The amount of displacement experienced by the all-suture anchor in the non-decorticated specimens was significantly greater at 10 cycles, 200 cycles, and 500 cycles (Table 1). The amount of displacement experienced by the all-suture anchor in the decorticated specimens was significantly greater at 10 cycles, 200 cycles, and 500 cycles (Table 2).

**Table 1:** Displacement with cyclic loading in the non-decorticated specimens.

Anchor	Displacement at 10 cycles (mm)	Displacement at 200 cycles (mm)	Displacement at 500 cycles (mm)
PEEK anchor	2.17, 0.96	2.48, 0.55	2.59, 0.57
All-suture anchor	4.06, 1.58	5.45, 2.08	5.75, 2.32
p-value	< 0.001	< 0.001	< 0.001

There was significantly more displacement with cyclic loading with the all-suture anchor at 10 cycles, 200 cycles, and 500 cycles. Data are presented as means, STD.

**Table 2:** Displacement with cyclic loading in the decorticated specimens.

Anchor	Displacement 10 cycles (mm)	Displacement 200 cycles (mm)	Displacement 500 cycles (mm)
PEEK anchor	2.57, 0.96	2.91, 1.04	3.03, 1.07
All-suture anchor	3.88, 2.15	5.60, 3.22	6.68, 3.83
p-value	0.004	< 0.001	< 0.001

There was significantly more displacement with cyclic loading with the all-suture anchor at 10 cycles, 200 cycles, and 500 cycles. Data are presented as means, STD.

The amount of displacement observed at the time of ultimate failure was significantly affected by anchor type for non-decorticated specimens (p = 0.01), but not for decorticated specimens (p = 0.08) (Table 3). With respect to ultimate load, anchor type did not show any significant difference (Table 4).

**Table 3:** Displacement at ultimate failure.

Anchor	Displacement at ultimate load (mm) (non-decorticated)	Displacement at ultimate load (mm) (decorticated)
PEEK anchor	10.5, 6.35	12.0, 7.36
All-suture anchor	15.3, 4.77	15.9, 5.38
p-value	0.01	0.08

There was significantly more displacement at the time of failure with all suture anchors for non-decorticated specimens, but not for decorticated specimens. Data are presented as means, STD.

**Table 4:** Ultimate failure load.

Anchor	Ultimate load (N) (non-decorticated)	Ultimate load (N) (decorticated)
PEEK anchor	220.9, 92.0	224.1, 84.5
All-suture anchor	182.3, 37.2	200.2, 40.4
p-value	0.33	0.71

There was no significant effect of anchor type on ultimate load. Data are presented as means, STD.

Both anchors had clinical and catastrophic failures in both the decorticated and non-decorticated cohorts. In the non-decorticated cohort, the PEEK anchors had 1 catastrophic failure and zero clinical failures (Table 5). The all-suture anchors had 2 catastrophic failures and 12 clinical failures. In the decorticated cohort the PEEK anchors had 2 catastrophic failures and 2 clinical failures (Table 6). The all-suture anchors had 3 catastrophic failures and 12 clinical failures.

**Table 5:** Non-decorticated data.

Anchor	Total failures N (%)	Catastrophic failures N (%)	Clinical Failure N (%)
PEEK Anchor	1/27 (3.7%)	1/27 (4%)	0/27 (0%)
All-suture anchor	14/27 (51.9%)	2/27 (7%)	12/27 (44%)

Catastrophic failure is anchor pull out prior to completion of cyclic loading. Clinical failure is defined as cyclic displacement greater than 5mm.

**Table 6:** Decorticated data.

Anchor	Total failures N (%)	Catastrophic failures N (%)	Clinical Failure N (%)
PEEK Anchor	4/27 (14.8%)	2/27 (7%)	2/27 (7%)
All-suture anchor	15/27 (55.6%)	3/27 (11%)	12/27 (44%)

Catastrophic failure is anchor pull out prior to completion of cyclic loading. Clinical failure is defined as cyclic displacement greater than 5mm.

The anterior region of the acetabulum consistently demonstrated the lowest bone density (p<0.0001) on CT-assisted osteodensitometry across all matched specimens (Table 7). There were no significant differences between non-decorticated and decorticated sides (p = 0.16).

**Table 7:** Bone Mineral Density (BMD) data.

	BMD (HU) (non-decorticated)	BMD (HU) (decorticated)
Superior	240.8, 101.1	244.9, 78.3
Anterosuperior	223.2, 63.8	250.7, 83.9
Anterior	81.1, 50.0	114.8, 79.3

There was no significant difference in bone mineral density between decortication groups. Data are presented as means, STD.

## Discussion

This study evaluated the cyclic displacement and ultimate load to failure of a commercially available all-suture anchor and solid PEEK anchor in human cadaveric acetabula. The PEEK anchor had significantly less displacement with cyclic loading both before and after decortication compared to the all-suture anchor. There was no significant difference in ultimate load to failure between the two anchors tested.

Acetabular labral tears are commonly repaired using suture anchors [12]. Smaller anchors have been developed to minimize bone loss while allowing more anchors to be used for fixation. In the hip, a smaller anchor reduces the risk of articular surface and psoas tunnel perforation [13, 14]. One of these smaller anchors is the all-suture anchor. While the all-suture anchor is smaller and preserves bone, if enough force isn’t used and the anchor is not fully seated against the cortical bone when deployed it will slip through the cancellous bone with cyclic loading [15].

In a bovine and human glenoid model, Dwyer et al. compared the biomechanical performance of a solid anchor and an all-suture anchor [16]. When the all-suture anchor was deployed by hand it demonstrated early displacement with cyclic loading. During the course of the study, they were able to determine that when the all-suture anchor was pre-tensioned to 60N, the early displacement with cyclic loading no longer occurred. Indicating that when the all-suture anchors were being deployed by hand, not enough force was being applied to seat the anchor against the cortical bone. This is consistent with the results of the current study in which the all-suture anchors were deployed by hand. In both the decorticated and non-decorticated cohorts, the all-suture anchors had greater than 4 mm of displacement in the first 10 cycles of loading. This likely represents the all-suture anchor slipping through the cancellous bone until it seats against the cortical surface during the first 10 cycles of loading.

In a study comparing multiple all-suture anchors and a single PEEK anchor, Douglass et al. evaluated these anchors in synthetic bone blocks of varying density [17]. The higher density synthetic bone block was selected to represent the bone density of the acetabulum. The lower density synthetic bone block was selected to represent the bone density of the glenoid. In general, the all-suture anchors performed better in the higher density bone model. This is because the all-suture anchor is less likely to slip through higher density cancellous bone until seated against the cortex, resulting in less displacement with cyclic loading. Similar to the results of the current study, they found the solid PEEK anchor to outperform all but one all-suture anchor in the higher density bone models.

In a biomechanical study, Ruiz-Suarez et al. evaluated six solid anchors along the acetabular rim [10]. Five of the six anchors tested were indicated for acetabular labral repair. Similar to the current study, they found the majority of displacement occurred within the first 100 cycles for five of the six anchors tested. One anchor tested had twice as much displacement after 500 cycles than it did after 100 cycles. In the current study, both the all-suture and PEEK anchor had over 50% of the displacement occur within the first 10 cycles. Unlike in their study, where no anchor displaced further than 2 mm, both anchors tested in current study had displacement greater than 2 mm at 200 and 500 cycles. This finding held true in both the non-decorticated and decorticated cohorts.

There are little data on the clinically relevant load to failure of the acetabular labrum. The normal load experienced by the acetabular labrum during walking is 40 N and 90 N during jogging [18]. Both anchors in the current study, in both the non-decorticated and decorticated cohorts and mean load to failures much higher than those experienced during jogging. As the site of failure is likely at the suture-labrum/capsule interface, displacement with cyclic loading is likely a more clinically relevant measure of the performance of a suture anchor for acetabular labrum repair [17].

In the setting of FAI, where acetabuloplasty routinely removes 1–2 mm of dense cortical bone prior to labral repair, it is unknown the effect that this has on the biomechanical performance of suture anchors [19, 20]. Both all-suture and solid anchors used for rotator cuff repair have been shown to have significantly lower ultimate load to failure after greater tuberosity decortication [21, 22]. In the current study, decortication didn’t significantly affect displacement with cyclic loading or load to failure in either of the anchors tested.

There is much debate as to the cause of failed arthroscopic labral repair in patients with persistent groin pain or microinstability. Potential culprits include strength of the initial labral repair, failure to heal, re-tear and capsular instability. While difficult to say with certainty, cyclic displacement and early gap formation at the labral repair-bone interface could be a contributor to the observed clinical findings. In the current study the all-suture anchor reached the 5-mm displacement threshold 44% of the time.

There were several limitations in the current study. Whenever possible, the placement of anchors was done in such a way as to mimic operative conditions, but there were several differences. The cadaveric specimens were stripped of all soft tissue and potted prior to anchor placement. This allowed for clear visualization of the region of anchor placement and enabled precise and reproducible angles of anchor insertion. A curved angle guide identical to that used intraoperatively was used to replicate operative conditions, but the actual placement of anchors during a case is much more difficult secondary to the presence of soft tissue, extent of muscle relaxation and various parameters of visualization inherent to any arthroscopic procedure. Further, the uniaxial test that was employed provided a consistent means of mechanically evaluating the pullout strength of the anchors; however, this did not accurately replicate the more complex in vivo loading. Additionally, the use of cadaveric specimens with an average age of 51 years, may not be indicative of that of the younger population generally undergoing such procedures.

Early displacement was higher with the all-suture anchors, while failure to load was equivalent. This may represent less stability of the all-suture anchor or that it requires more early tension to “set” the anchor. Clinically, this may not be relevant, given the lower loads on the labrum and repair with normal activities.

## Supporting information

S1 Data (XLSX)
